# Defect-to-Phase Transduction and Interface Reconstruction Enable Regeneration of Spent LiFePO_4_ Cathodes

**DOI:** 10.1007/s40820-026-02451-x

**Published:** 2026-11-06

**Authors:** Liwei Chen, Zhiheng Wu, Shouyu Qu, Siyu Zhang, Magdi E. A. Zaki, Mohamed H. Helal, Yangyang Liu, Guanhua Jin, Jiang Zhou

**Affiliations:** 1 Xinjiang Key Laboratory of New Energy and Energy Storage Technology, Xinjiang Institute of Technology, Aksu, 843100 Xinjiang People’s Republic of China; 2 School of Materials Science and Engineering, Hunan Provincial Key Laboratory of Electronic Packaging and Advanced Functional Materials, Central South University, Changsha, 410083 People’s Republic of China; 3 Department of Chemistry, Faculty of Science, Imam Mohammad Ibn Saud Islamic University (IMSIU), 11623 Riyadh, Saudi Arabia; 4 Center for Scientific Research and Entrepreneurship, Northern Border University, 73213 Arar, Saudi Arabia; 5 School of Instrument Science and Technology, Xi’an Jiaotong University, Xi’an, 710049 People’s Republic of China

**Keywords:** Spent LiFePO_4_ cathodes, Direct regeneration, Defect-to-phase transduction, Lattice reconstruction, Anti-site defects

## Abstract

Retired lithium-ion batteries are becoming an important material resource, yet recovering cathode value without dissolution and resynthesis remains challenging. Direct regeneration of spent LiFePO_4_ is hindered by Li vacancies, Li-Fe anti-site defects, and heterogeneous Fe valences trapped within the olivine lattice. Here we examine defect-to-phase transduction together with carbon-interface reconstruction to address this constraint. Controlled oxidation converts the detectable olivine phase into intermediates containing Li_3_Fe_2_(PO_4_)_3_ and Fe_2_O_3_, removing the inherited olivine site topology. Lithiation and reduction subsequently reconstruct LiFePO_4_ from these intermediates. Rietveld refinement estimates a decrease in Fe-on-Li site occupancy from 5.72% to 0.68%, with a further estimated decrease to 0.35% in the guanidine-assisted sample and a narrower measured electrochemical response. The regenerated cathode delivers 167 mAh g^-1^ at 0.1 C and 123 mAh g^-1^ at 5 C, retaining 99.2% capacity after 500 cycles at 1 C. Process analysis estimates a profit of US$2.28 kg^-1^ of spent cell recycled with lower energy use and greenhouse gas emissions than hydrometallurgical recycling. These results link phase reconstruction and carbon-interface regulation with structural recovery and improved electrode response in spent LiFePO_4_.

**Supplementary Information:**

The online version contains supplementary material available at https://doi.org/10.1007/s40820-026-02451-x.

## Highlights

- Defect-to-phase transduction describes oxidative conversion of the degraded olivine lattice into intermediates from which LiFePO_4_ is reconstructed.
- Guanidine hydrochloride supplies nitrogen during carbon formation, constructing an N-doped carbon interface associated with improved electrochemical response and lower refined Fe-on-Li site occupancy.
- This coupled lattice-interface reconstruction yields low refined Fe-on-Li site occupancy, high-rate performance and durable cycling in regenerated spent LiFePO_4_ cathodes.

**Supplementary Information:**

The online version contains supplementary material available at https://doi.org/10.1007/s40820-026-02451-x.

## Graphical abstract

**Figure:**
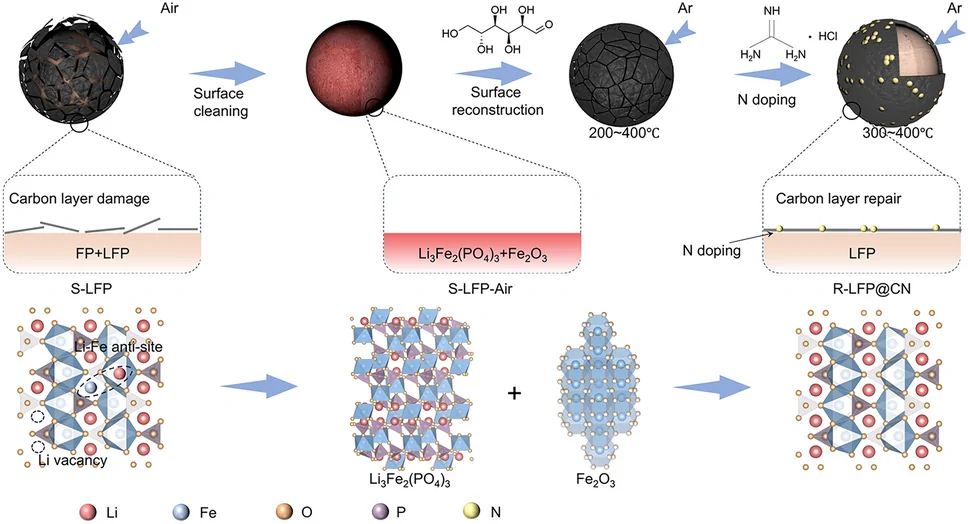

## Introduction

The rapid expansion of lithium-ion batteries is creating a growing stream of retired cathodes that retain substantial elemental, structural, and manufacturing value [1]. This issue is particularly important for LiFePO_4_ (LFP), whose widespread use in electric vehicles and stationary storage will generate large quantities of spent material [2–7]. Hydrometallurgical and pyrometallurgical recycling recover elemental resources but sacrifice the crystal structure and particle architecture created during cathode production [8, 9]. Direct regeneration seeks to preserve these assets by restoring the active material in the solid state, thereby retaining value that elemental recovery cannot capture [10–13]. Its success, however, requires more than replenishing lithium (Li). Spent LFP (S-LFP) contains coupled Li vacancies, Li-Fe anti-site defects, FePO_4_ (FP) domains, heterogeneous Fe valences, and surface-to-bulk structural gradients that remain kinetically trapped in the olivine lattice [14–17]. Restoring bulk composition alone cannot ensure recovery of the ordered structure.

Many direct regeneration methods prolong thermal treatment, introduce reducing agents, or rebuild conductive carbon [18–20]. Such treatments can refill Li vacancies and reduce Fe^3+^, but when the parent phase is retained, correction of site disorder still requires cation redistribution within that framework. Fe occupying a Li site must still migrate through the crystal and return to the Fe sublattice. The same anti-site blocks the one-dimensional Li channels required for uniform relithiation, creating a self-reinforcing kinetic constraint. This limitation becomes more severe as degradation penetrates the particle and different defect populations become spatially coupled. Thermal treatment may promote cation redistribution but also accelerates particle coarsening, sintering, and carbon degradation. Complete dissolution eliminates inherited site occupancy, although it abandons the structural and energetic advantages of direct regeneration. Oxidation-assisted homogenization and confined repair have already demonstrated intermediate-phase formation followed by olivine reconstruction [19]. The remaining question addressed here is how lattice recovery and carbon-interface modification contribute to residual site disorder and the regenerated electrode response.

An alternative begins by recognizing that a point defect is not an autonomous entity but a deviation from a crystal lattice. Once a reconstructive phase transition replaces that lattice, the distinction between a vacant Li site and one occupied by Fe ceases to exist. The original site definitions are removed with the parent topology, although residual disorder can form during reconstruction. We describe this change in the object of regeneration as defect-to-phase transduction. Elemental inventories pass into intermediate phases, from which the target lattice is rebuilt by recombination. The viability of this route depends on how those phases form and evolve. They must retain the elements required for the target compound and allow mass redistribution during reconstruction, while lithium and electrons must reach the reaction front before coarsening or separation intervenes. This interpretation distinguishes reconstruction from repair within a retained lattice, while its microscopic transport pathway and complete phase balance require evidence beyond endpoint phase identification.

Building on oxidation-assisted regeneration, we examined an oxidation and reconstruction route for S-LFP. Controlled air oxidation transforms the degraded lattice into Li_3_Fe_2_(PO_4_)_3_ and Fe_2_O_3_, after which Li compensation and reduction regenerate LFP from the identified intermediates. Guanidine-derived nitrogen-doped carbon supports the reverse reconstruction. To verify the effect, the regenerated sample prepared without guanidine hydrochloride (CH_6_ClN_3_) during annealing was designated R-LFP. The guanidine-free material shows a decrease in refined Fe-on-Li site occupancy from 5.72% to 0.68%, with an estimated value of 0.35% for the nitrogen-doped carbon sample. The regenerated R-LFP@CN delivers 167 mAh g^-1^ at 0.1 C and 123 mAh g^-1^ at 5 C, retaining 99.2% of its capacity after 500 cycles at 1 C. Process analysis also indicates economic and environmental advantages over hydrometallurgical recycling. This comparison examines structural recovery along the complete regeneration route and the additional changes associated with guanidine-assisted carbon-interface reconstruction.

## Experimental Section

### Materials

All reagents were of analytical grade and used as received. Guanidine hydrochloride (CH_6_ClN_3_, AR, ≥99.9%), glucose (C_6_H_12_O_6_, AR, ≥99.0%), lithium carbonate (Li_2_CO_3_, AR, ≥99.0%) and N-methyl-2-pyrrolidone (NMP, AR, ≥99.0%) were purchased from Aladdin (Shanghai, China). 1 M LiPF_6_ in ethylene carbonate (EC): diethyl carbonate (DEC): dimethyl carbonate (DMC) (1:1:1 in volume) as electrolyte (LB-014) was purchased from DoDochem (Suzhou, China). Lithium metal disks were purchased from Guangdong Canrd New Energy Technology Co. Ltd.

### Battery Disassembly and Separation

The spent LFP cell was first immersed in a 1 M NaCl solution for 24 h to ensure complete discharge. It was then manually disassembled to separate the cathode electrode, anode electrode, aluminum plastic film, and separator. The cathode electrode was crushed and sieved to obtain the S-LFP active material powder.

### Preparation of S-LFP-Air, R-LFP, and R-LFP@CN

S-LFP was pre-oxidized in a muffle furnace at 500 °C for 2 h in air, and the resulting powder was denoted S-LFP-Air. For the preparation of R-LFP@CN, S-LFP-Air was mixed with glucose and guanidine hydrochloride at 12 and 2.5 wt% relative to the mass of the initial S-LFP. Li_2_CO_3_ was added to supply supplementary Li at a nominal Li/Fe molar ratio of 25:100. The mixture was ball-milled at 500 r min^-1^ for 2 h and then sieved. The mixed precursor was transferred to a tube furnace and heated under flowing Ar. The temperature was increased to 400 °C at 5 °C min^-1^ and held for 4 h, followed by heating to 700 °C at 5 °C min^-1^ and holding for 6 h. The furnace was then allowed to cool naturally to room temperature under Ar, yielding R-LFP@CN. The control sample R-LFP was prepared using the same procedure but without guanidine hydrochloride.

### Material Characterization

The elemental composition of the cathode materials was determined by Inductively Coupled Plasma Optical Emission Spectrometry (ICP-OES, Agilent 720ES(OES), USA). Phase structure and model-derived Fe-on-Li site occupancy were analyzed by X-ray diffraction (XRD, Rigaku Smartlab, Japan) using Cu Kα radiation over a 2θ range of 10°-80° (scanning rate: 2° min^-1^). The degree of graphitization of carbon on the particle surface was characterized by Raman spectroscopy (HORIBA LabRAM HR800 France). Changes in the local phosphate environment were also examined by Fourier transform infrared spectroscopy (FT-IR, Nicolet 6700, USA). The morphology, microstructure, and elemental distribution of the samples were observed using scanning electron microscopy (SEM, FEI Nova NanoSEM, USA) and transmission electron microscopy (TEM, Talos F200X, USA). The near-surface chemical states of the elements were investigated by X-ray photoelectron spectroscopy (XPS, Thermo Fisher-K-Alpha, USA).

### Electrochemical Performance Tests

The cathode powder, acetylene black, and PVDF binder were mixed at a weight ratio of 8:1:1. A homogeneous slurry was formed in an NMP solution, coated onto aluminum foil, and dried in a vacuum oven at 80 °C for 12 h. The cathode coating areal density is 0.88-1.76 mg cm^-2^. The CR2025 coin cells were assembled in an argon-filled glove box. The charge–discharge performance was evaluated using LAND and NEWARE battery test systems within a voltage range of 2.2-4.2 V versus Li^+^/Li. Cyclic voltammetry (CV) and electrochemical impedance spectroscopy (EIS) measurements were conducted on a Corrtest electrochemical workstation at 25 °C. The CV scan rates ranged from 0.1 to 1.0 mV s^-1^.

## Results and Discussion

### Defect-to-Phase Transduction and Lattice Reconstruction

**Fig. 1:**
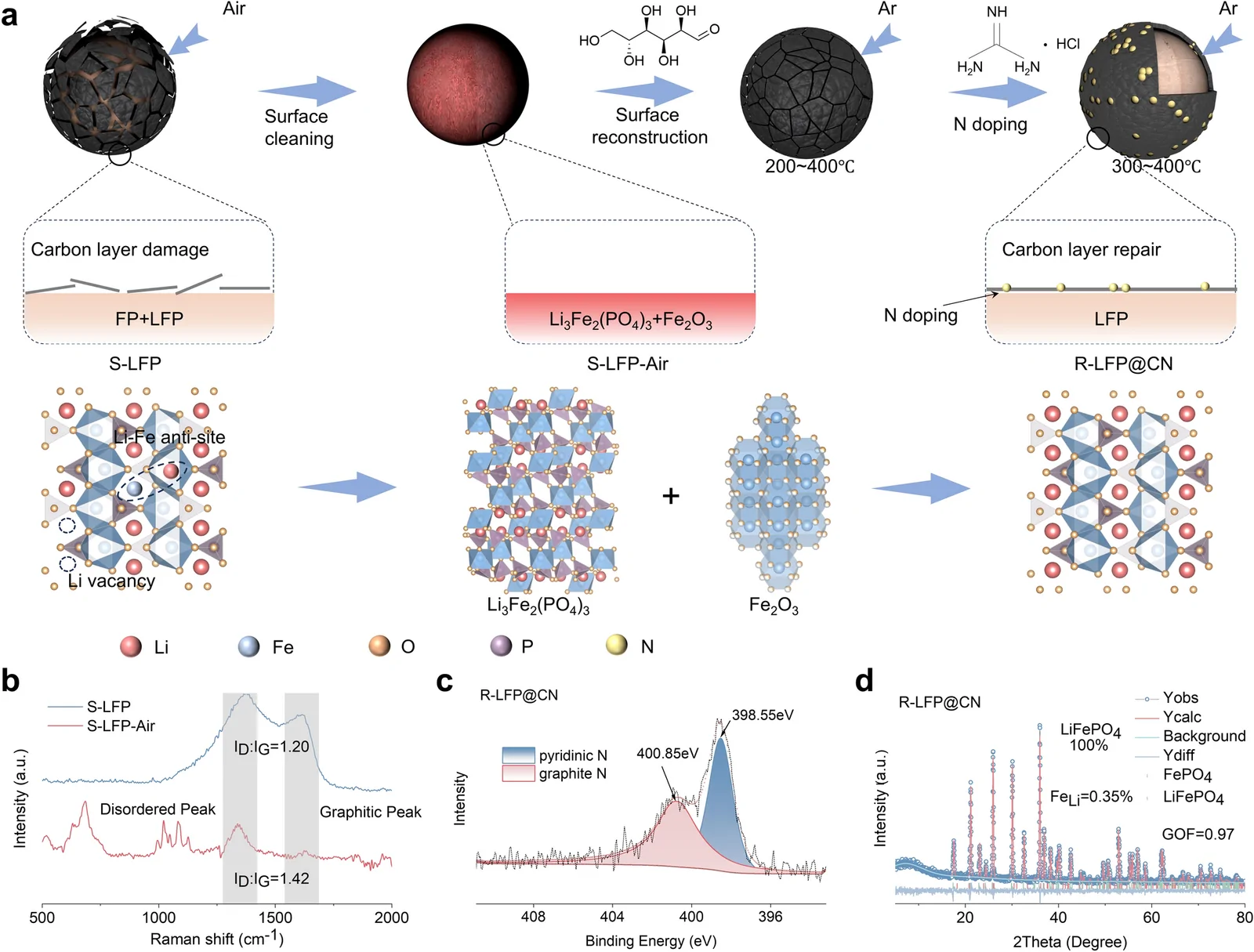
Defect-to-phase transduction and reconstruction of the LiFePO_4_ lattice and carbon interface. **a** Schematic illustration of the regeneration pathway. **b** Raman spectra of S-LFP and S-LFP-Air showing attenuation and increased disorder of residual surface carbon after oxidation. **c** High-resolution N 1*s* XPS spectrum of R-LFP@CN, resolved into pyridinic N at 398.55 eV and graphitic N at 400.85 eV. **d** Rietveld refinement of the XRD pattern of R-LFP@CN; Fe-on-Li site occupancy is a model-derived estimate

S-LFP exhibits coupled degradation of the olivine lattice and its carbon-coated interface. Li vacancies, Li-Fe anti-site defects, and FP domains accumulate within the particles, while the conductive carbon fragments and residual polyvinylidene fluoride remain at the surface. Repairing either region alone would leave an important source of failure unresolved. Figure 1a presents a defect-to-phase transduction route that converts the detectable olivine phase into intermediates before rebuilding the lattice and interface. S-LFP was pre-oxidized at 500 °C in air, then mixed with Li_2_CO_3_ and carbon/nitrogen sources and annealed at 700 °C under Ar to yield R-LFP@CN. The ideal oxidation relation 6LiFePO_4 _+ 3/2 O_2_ → 2Li_3_Fe_2_(PO_4_)_3 _+ Fe_2_O_3_ identifies compositionally complementary intermediates. This relation applies to fully lithiated LFP; the Li-deficient feedstock requires separate Li compensation, and XRD alone does not establish its complete intermediate-phase balance. After Li compensation, reductive annealing regenerates LFP from the identified intermediates. Air oxidation at 500 °C establishes the intermediate state required for reconstruction. LFP reflections disappear while Li_3_Fe_2_(PO_4_)_3_ and Fe_2_O_3_ emerge in Fig. S1, showing conversion of the detectable olivine phase without excluding residual or noncrystalline components. Surface Fe changes from mixed Fe^2+^ and Fe^3+^ in S-LFP to Fe^3+^ in S-LFP-Air in Fig. S2. As shown in Fig. 1b, the G band is strongly attenuated, and the residual I_D_ to I_G_ ratio increases from 1.20 to 1.42, consistent with extensive removal of the original carbon layer. The band near 1400 cm^-1^ associated with residual polyvinylidene fluoride is no longer resolved in Fig. S3. Pre-oxidation therefore changes the detected crystalline phase assemblage and substantially attenuates the damaged surface carbon and binder signatures before reconstruction.

Thermogravimetric analysis provides a basis for the annealing program. Glucose (C_6_H_12_O_6_) decomposes mainly between 200 and 400 °C, with its fastest mass loss between 200 and 300 °C, while CH_6_ClN_3_ reaches its maximum decomposition rate near 330 °C (Fig. S4). The overlap  of their decomposition ranges between 300 and 400 °C suggests a thermal interval for combined carbonization and nitrogen-precursor conversion, as supported by the precursor mixture (Fig. S5). Holding at 400 °C was selected to promote initial carbonization within this overlapping interval, while treatment at 700 °C drives Li_2_CO_3_-assisted relithiation, reduction and carbon ordering. The TG data do not independently identify the volatile nitrogen species or establish an optimum holding temperature. Chemical analysis tracks Li replenishment: Li/Fe increases from 0.812 in S-LFP to 1.040 in R-LFP@CN, while P/Fe changes from 0.961 to 0.966 (Table S1). These bulk ratios do not independently demonstrate exact stoichiometry or a complete phase balance. Fe 2*p* spectra show that the Fe^3+^-dominated S-LFP-Air surface returns to a predominantly Fe^2+^ state after annealing (Fig. S6). The residual C-F component disappears from both regenerated samples, whereas a C-N component emerges only in R-LFP@CN (Fig. S7). Pyridinic N at 398.55 eV and graphitic N at 400.85 eV are resolved in Fig. 1c; the undoped control spectra are shown in Fig. S8. These configurations may contribute to electronic transport and interfacial charge transfer [21–23], although their individual roles are not resolved by the present measurements.

X-ray diffraction (XRD) Rietveld refinement estimates structural parameters associated with regeneration. S-LFP contains 22.5% FP, whereas no FP is detected in R-LFP or R-LFP@CN (Figs. 1d and S9). The refined Fe-on-Li site occupancy decreases from 5.72% in S-LFP to 0.68% in R-LFP and 0.35% in R-LFP@CN. The large decrease without guanidine accompanies the complete oxidation, relithiation and reductive-annealing route. The lower refined value for R-LFP@CN is associated with guanidine-assisted carbon formation, but the difference between 0.68% and 0.35% is not independent proof of a nitrogen-specific defect-suppression mechanism. The PO_4_^3-^ vibration shifts from 1018.23 cm^-1^ in S-LFP to 1016.30 cm^-1^ in R-LFP and 1014.37 cm^-1^ in R-LFP@CN (Fig. S10), indicating a change in the local phosphate environment [24]. Carbon ordering follows the same hierarchy. The I_D_ to I_G_ ratio decreases from 1.20 in S-LFP to 1.14 in R-LFP, and 1.07 in R-LFP@CN (Fig. S11). The lower value for R-LFP@CN is consistent with a more ordered carbon framework but does not isolate the contribution of graphitic nitrogen. Together, oxidative phase conversion changes the parent phase assemblage, reductive relithiation reconstructs LFP, and guanidine-assisted carbon formation is associated with additional interfacial and structural changes. These endpoint results support lattice and interface recovery along the combined route; they do not isolate the effect of pre-oxidation from relithiation, carbon-assisted reduction and annealing.

### Recovery of Local Structure and Valence Profiles

**Fig. 2:**
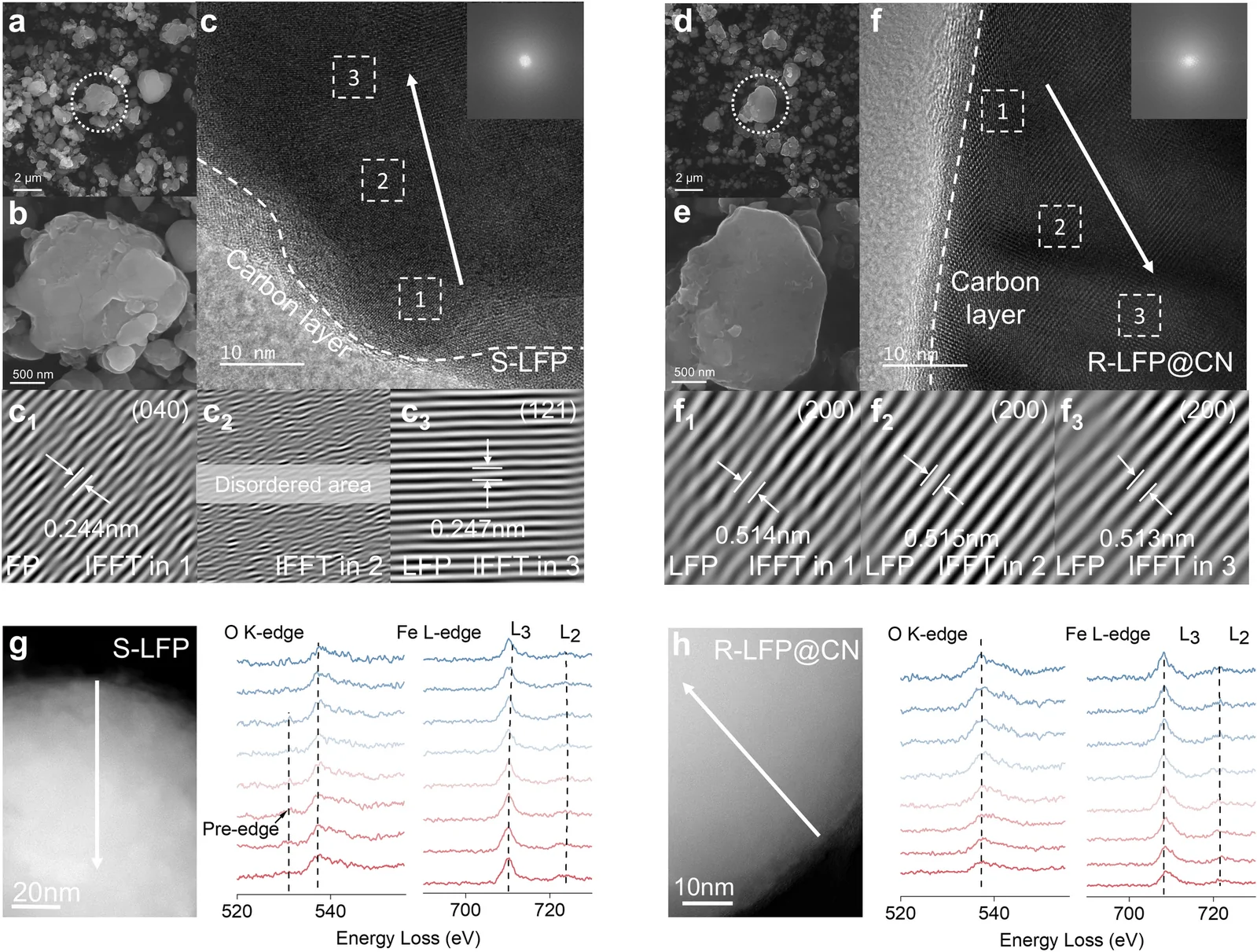
Local structural and valence restoration after regeneration. **a** SEM image of S-LFP showing agglomerated spent particles. **b** High-magnification SEM image revealing surface microcracks in S-LFP. **c** TEM characterization of S-LFP; inverse fast Fourier transform images from the surface, intermediate region and particle interior are presented in c_1_-c_3_, respectively. **d** SEM image of R-LFP@CN showing a more uniform regenerated morphology. **e** High-magnification SEM image revealing a smoother reconstructed surface in R-LFP@CN. **f** TEM characterization of R-LFP@CN showing coherent LiFePO_4_ lattice fringes extending from the surface toward the interior; corresponding inverse fast Fourier transform images are presented in f_1_-f_3_. **g** EELS line scan across an S-LFP particle. **h** EELS line scan of R-LFP@CN showing no resolved O K-edge pre-edge feature and nearly invariant Fe *L*_2,3_-edge positions along the measured trajectory

Local structure and valence profiles were examined to assess whether regeneration reduces the heterogeneity observed in the spent particles. Scanning electron microscopy (SEM) first reveals the accompanying morphological changes. S-LFP consists of strongly agglomerated particles, whereas R-LFP@CN shows regenerated particles with a smoother apparent surface morphology (Fig. 2a, d). At higher magnification, microcracks are evident on the S-LFP surface, while the regenerated particles present a smoother and more continuous exterior (Fig. 2b, e). This contrast is consistent with removal of the damaged surface residues followed by reconstruction of the carbon interface. Elemental maps of Fe, O and P for S-LFP and R-LFP@CN are shown in Fig. S12. Transmission electron microscopy (TEM) provides the required structural resolution. S-LFP shows a diffuse diffraction pattern in the inset (Fig. 2c), indicating disrupted crystallographic coherence. Inverse fast Fourier transform analysis further resolves a pronounced surface-to-bulk gradient. Region 1 at the particle surface shows a lattice spacing of 0.244 nm. Its interpretation as an FP-enriched surface region is considered together with the EELS evidence. Region 2 contains disordered fringes consistent with local structural heterogeneity, whereas Region 3 in the interior exhibits a spacing of 0.247 nm corresponding to the (121) plane of LFP in Fig. 2c. The differences between the surface, intermediate region and particle interior are consistent with a surface-enriched degraded state and local structural heterogeneity. Because FP and LFP can exhibit similar lattice spacings, a single fringe spacing does not independently establish phase identity. R-LFP@CN displays a markedly sharper diffraction pattern and continuous lattice fringes from the exterior to the interior (Fig. 2f). The measured spacing of 0.514 nm corresponds to the (200) plane of LFP, supporting reconstruction of a coherent olivine lattice across the examined particle region.

Electron energy loss spectroscopy (EELS) is used to determine whether structural homogenization is accompanied by recovery of the Fe chemical state. At the S-LFP surface, the O K-edge contains a pronounced pre-edge feature associated with Fe^3+^, while the Fe *L*_2,3_-edge occurs at higher energy loss (Fig. 2g). The pre-edge intensity progressively decreases toward the particle interior and ultimately disappears, accompanied by a shift of the Fe *L*_2,3_-edge to lower energy loss. These correlated changes identify a surface-enriched Fe^3+^ state that evolves toward Fe^2+^ in the interior, in agreement with the FP-to-LFP structural gradient. By contrast, R-LFP@CN shows no resolved O K pre-edge feature along the measured trajectory, and the Fe *L*_2,3_-edge remains at a nearly constant position (Fig. 2h). The regenerated particle is therefore predominantly Fe^2+^ and chemically more uniform along the line scan direction. Thus, the examined, spent and regenerated particles differ in their local structural and valence profiles. These observations support local restoration of the olivine structure and Fe^2+^ state, while the microscopic reconstruction pathway remains inferred from the phase measurements.

### Electrochemical Response after Lattice and Interface Reconstruction

**Fig. 3:**
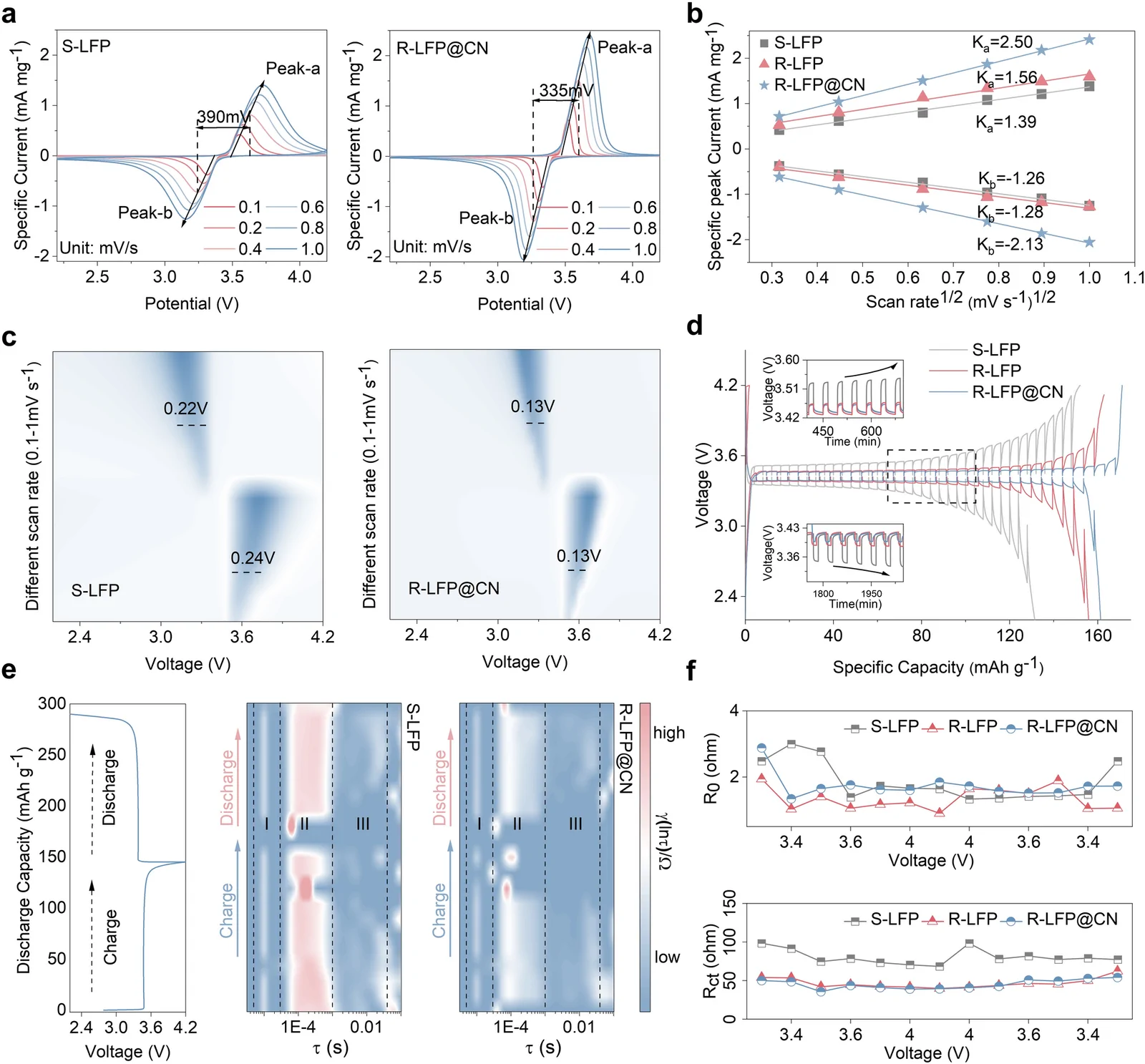
Electrochemical response following lattice and interface reconstruction.** a** Cyclic voltammograms of S-LFP and R-LFP@CN. **b** Linear dependence of mass-normalized peak current on the square root of scan rate between 0.1 and 1.0 mV s^-1^. **c** Cyclic voltammetry contour maps showing the distribution, symmetry and width of the redox responses. R-LFP@CN exhibits a narrower and more symmetric response than S-LFP. **d** Galvanostatic intermittent titration profiles demonstrating the lower transient polarization of the regenerated materials. **e** In situ distribution-of-relaxation-times maps. **f** Fitted ohmic resistance (R_0_) and charge transfer resistance (R_ct_) during charge and discharge. Both regenerated samples show lower R_ct_ and smaller voltage-dependent variations than S-LFP

The reconstructed electrodes were examined for changes in polarization and the breadth of the ensemble electrochemical response. Cyclic voltammetry (CV) provides the first test of this expectation. All three electrodes show the Fe^2+^ and Fe^3+^ redox couple in Figs. 3a and S13. At 0.4 mV s^-1^, the peak separation decreases from 390 mV for S-LFP to 315 mV for R-LFP and 335 mV for R-LFP@CN. Both regenerated samples therefore show lower polarization than S-LFP. R-LFP has a slightly smaller separation than R-LFP@CN, demonstrating that guanidine addition does not improve every kinetic descriptor and that the guanidine-free regeneration route already recovers substantial reversibility. Scan rate-dependent measurements from 0.1 to 1 mV s^-1^ are discussed qualitatively using the Randles–Sevcik relation [14]:

1 $$I_{p} = \left( {2.69 \times 10^{5} } \right)n^{{{\raise0.7ex\hbox{$3$} \!\mathord{\left/ {\vphantom {3 2}}\right.\kern-\nulldelimiterspace} \!\lower0.7ex\hbox{$2$}}}} SD_{{Li}}^{{{\raise0.7ex\hbox{$1$} \!\mathord{\left/ {\vphantom {1 2}}\right.\kern-\nulldelimiterspace} \!\lower0.7ex\hbox{$2$}}}} C_{{Li}} v^{{{\raise0.7ex\hbox{$1$} \!\mathord{\left/ {\vphantom {1 2}}\right.\kern-\nulldelimiterspace} \!\lower0.7ex\hbox{$2$}}}}$$

Equation (1) uses absolute peak current and a scan rate expressed in V s^-1^; n is the electron-transfer number, S the electrode area, D_Li_ the diffusion coefficient, and C_Li_ the concentration term in the conventional relation. Because Fig. 3b uses mass-normalized peak current, its slopes are compared qualitatively rather than converted directly into intrinsic Li^+^ diffusion coefficients. Figure 3b shows slopes in the order S-LFP < R-LFP < R-LFP@CN, indicating a larger mass-normalized CV response for R-LFP@CN. CV contour plots further compare the breadth and symmetry of the redox responses. S-LFP shows asymmetric peaks with full widths at half maximum of 0.24 V during oxidation and 0.22 V during reduction (Figs. 3c and S14). Such broadening is consistent with a broader ensemble response to which FP domains, anti-site defects, and the surface-to-bulk degradation gradient can contribute. The regenerated electrodes show more symmetric responses, and R-LFP@CN exhibits the narrowest and most closely matched oxidation and reduction peaks. Regeneration is therefore associated with a narrower electrode-level redox response, which does not independently quantify the spatial distribution of local reaction rates.

Galvanostatic intermittent titration (GITT) measurements support this interpretation (Fig. 3d). S-LFP develops the largest transient overpotentials during both lithiation and delithiation, consistent with hindered Li transport through a spent material with a refined Fe-on-Li site occupancy of 5.72%. These defects obstruct the [010] channels, but the measured overpotential cannot be assigned to anti-site defects alone because phase heterogeneity and the damaged interface also contribute [25]. R-LFP shows lower polarization after removal of FP and reduction of the anti-site fraction, while R-LFP@CN gives the smallest transient response. Its improvement is consistent with the combination of a reconstructed bulk lattice and a more ordered conductive coating. Graphitic and pyridinic nitrogen may further assist electronic and interfacial transport, although the present electrochemical data do not independently quantify their individual contributions. In situ electrochemical impedance spectroscopy resolves the interfacial component of this recovery. Both regenerated samples show lower fitted charge transfer resistance than S-LFP, with comparable values and some crossings between R-LFP and R-LFP@CN across the measured states of charge (Figs. 3f and S15). Distribution of relaxation times (DRT) analysis separates the impedance response according to characteristic time constants. The intermediate frequency feature in Region II is tentatively associated with coupled interfacial and charge transfer processes. S-LFP displays a broad and intense Region II response in Fig. 3e, consistent with a more dispersed relaxation response. R-LFP gives a weaker intermediate response (Fig. S16), while R-LFP@CN shows the lowest intensity and narrowest distribution [26]. This progression accompanies structural and interfacial recovery, although the DRT features do not uniquely resolve the microscopic processes or spatial reaction-rate distribution.

Taken together, the electrochemical measurements reveal a hierarchy consistent with the structural evidence. The complete regeneration route restores electrochemically accessible LFP and lowers polarization. Guanidine-assisted carbon-interface reconstruction is associated with a narrower electrochemical response, although *R*_ct_ does not consistently rank R-LFP@CN below R-LFP. The comparison supports improved ensemble response without independently establishing spatially uniform reaction rates.

### Electrochemical Persistence of the Reconstructed State

**Fig. 4:**
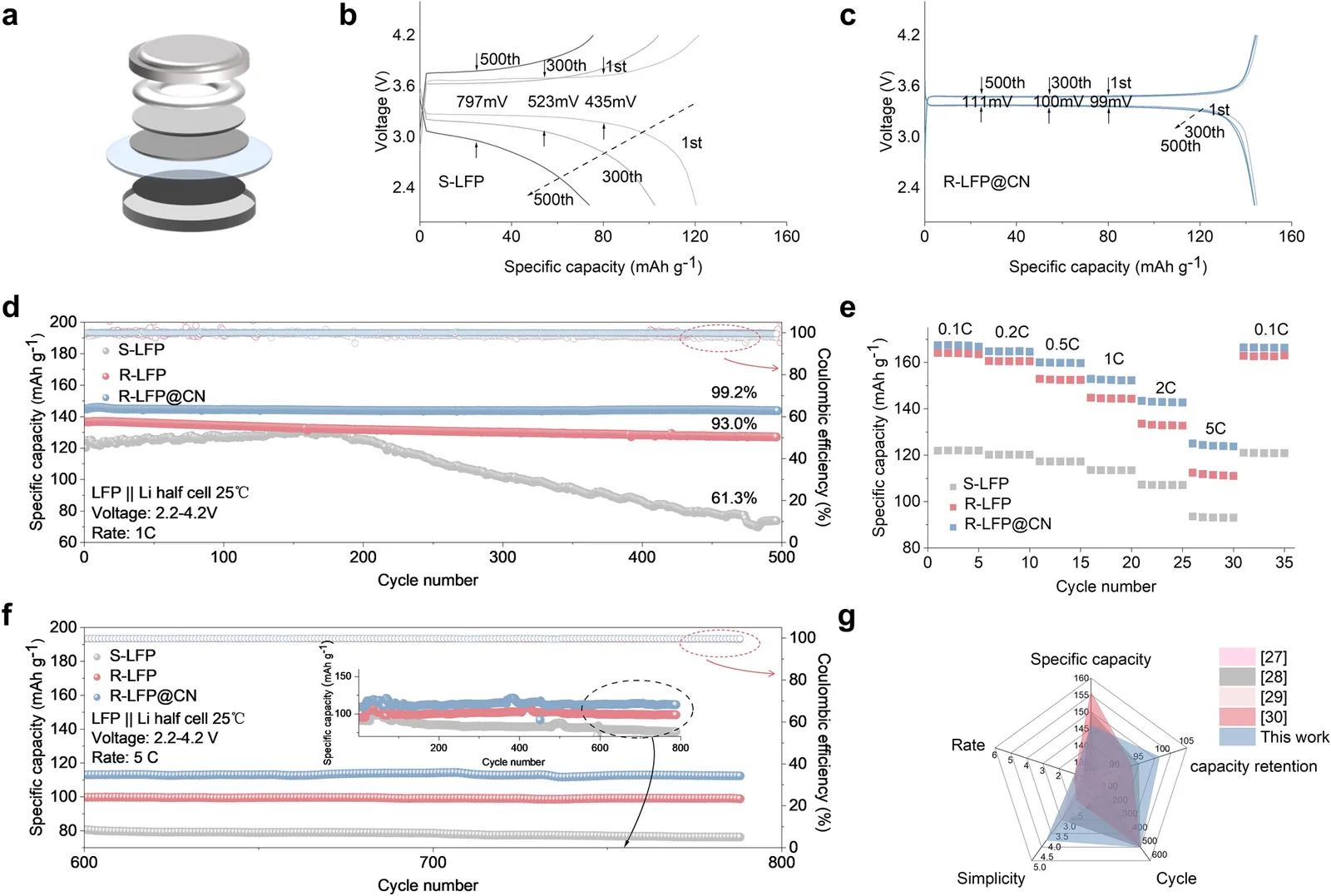
Electrochemical persistence of reconstructed LiFePO_4_.** a** Schematic configuration of the LFP-Li half-cell used for electrochemical evaluation. **b, c** Galvanostatic charge and discharge profiles of S-LFP and R-LFP@CN at 1 C during the first, 300th and 500th cycles. The marked values represent the voltage separation between the charge and discharge plateaus. **d** Cycling performance and Coulombic efficiency at 1 C between 2.2 and 4.2 V at 25 °C. After 500 cycles, S-LFP, R-LFP and R-LFP@CN retain 61.3%, 93.0% and 99.2% of their initial capacities, respectively. **e** Rate performance from 0.1 to 5 C followed by recovery at 0.1 C. R-LFP@CN delivers 167 mAh g^-1^ at 0.1 C and 123 mAh g^-1^ at 5 C. **f** Long-term cycling at 5 C over 800 cycles. **g** Illustrative comparison of specific capacity, capacity retention, cycling duration, rate capability and process simplicity with reported LFP regeneration strategies. Process simplicity is a qualitative descriptor rather than a calibrated performance metric

The electrochemical durability of regeneration is assessed by whether the reconstructed LFP state remains electrochemically accessible during prolonged cycling. Li half-cells were therefore assembled as shown in Fig. 4a. The charge and discharge profiles at 1 C immediately distinguish the spent and regenerated materials in Figs. 4b-c and S17. S-LFP delivers the lowest capacity, and its voltage separation increases from 435 mV in the first cycle to 523 mV after 300 cycles and 797 mV after 500 cycles. This progressive polarization is consistent with continued loss of reaction uniformity in a lattice containing FP, anti-site defects, and a damaged conductive interface. R-LFP@CN retains much flatter profiles, with corresponding voltage separations of 99, 100, and 111 mV. The limited increase over 500 cycles indicates that electrochemical access to the reconstructed lattice is maintained. Long-term cycling at 1 C provides a more stringent comparison in Fig. 4d. S-LFP retains only 61.3% of its initial capacity after 500 cycles, whereas R-LFP retains 93.0% and R-LFP@CN retains 99.2%. The substantial recovery already observed for R-LFP is consistent with removal of FP and reduction of Li-Fe anti-site defects along the complete oxidation, relithiation and reductive-annealing route.

The additional stability of R-LFP@CN is associated with the recovered lattice structure and modified carbon interface. This comparison identifies additional changes associated with guanidine-assisted interface formation within the same overall regeneration route. The same hierarchy persists as the current increases. R-LFP@CN delivers 167 mAh g^-1^ at 0.1 C and retains 123 mAh g^-1^ at 5 C (Fig. 4e). At 5 C, R-LFP remains below R-LFP@CN, while S-LFP delivers 93 mAh g^-1^. Capacity recovers when the rate returns to 0.1 C, showing that the high-current response is largely reversible rather than being caused by permanent structural damage. Extended cycling at 5 C further confirms that R-LFP@CN maintains the highest capacity and the most stable response over 800 cycles (Fig. 4f). These results agree with the structural and kinetic measurements. The reconstructed bulk lattice restores continuous Li transport pathways, while the more ordered nitrogen-containing carbon network supports electron transfer and is associated with a narrower ensemble electrochemical response. The present data support this combined contribution but do not isolate a unique transport role for each nitrogen configuration. Comparison with reported regeneration strategies places these results in a broader context. The illustrative radar plot in Fig. 4g compares specific capacity, rate capability, cycling duration, capacity retention, and process simplicity against representative studies [27–30]. The comparison provides literature context but does not establish an overall ranking across studies with different cycling and electrode conditions. Together, the oxidation, relithiation and reductive-annealing route restores LFP structure and electrode performance, with additional improvements associated with guanidine-assisted interface reconstruction. The rate capability and cycling stability demonstrate recovery under the tested Li half-cell conditions.

### Process Level Implications of Preserving Cathode Value

**Fig. 5:**
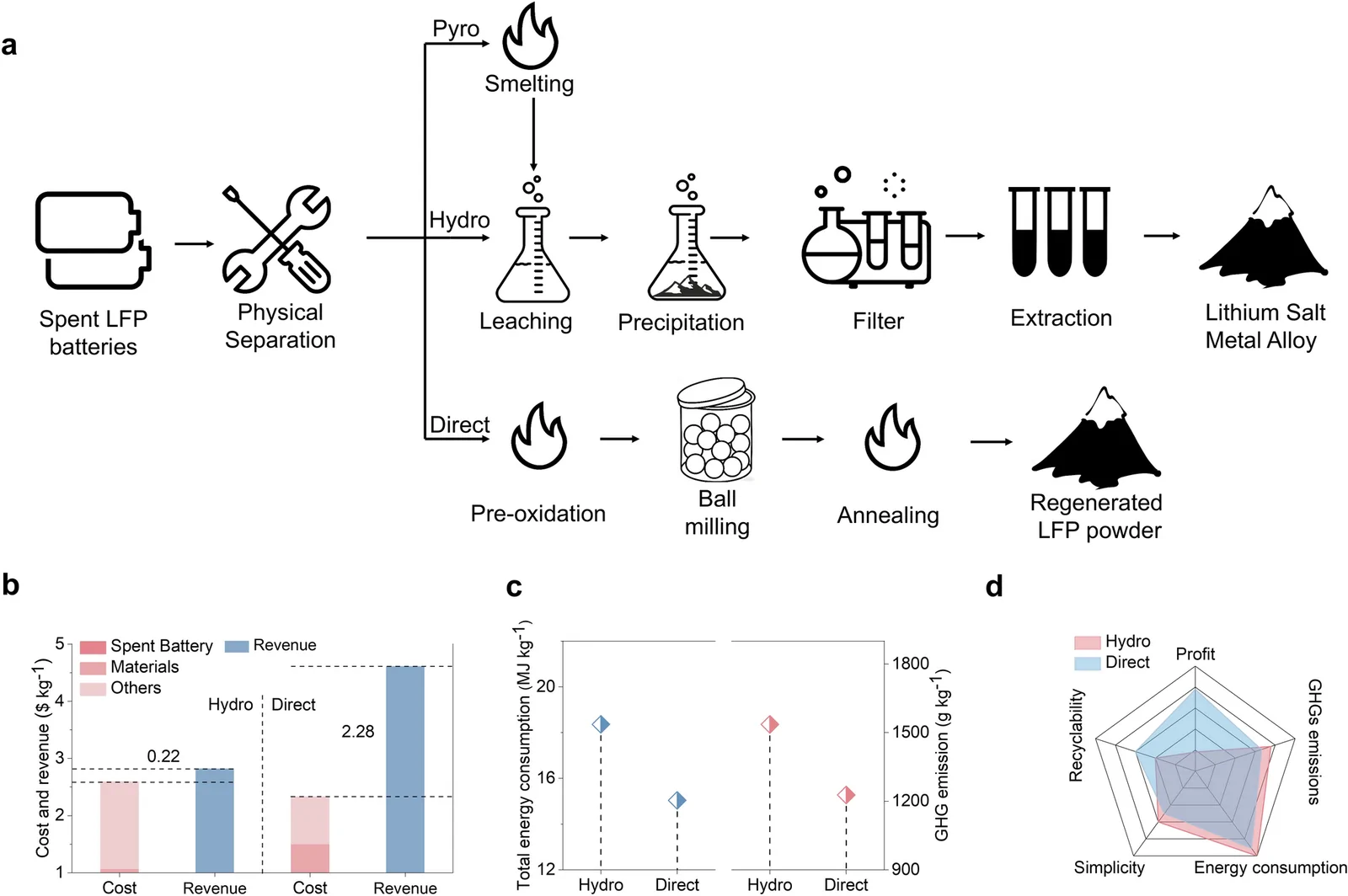
Modeled economic and environmental assessment of direct LiFePO_4_ regeneration.** a** Comparison of the principal material and processing flows for pyrometallurgical recycling, hydrometallurgical recycling and the proposed direct regeneration route.** b** Modeled net profit per kilogram of spent LFP cell recycled calculated using EverBatt 2023. **c** Modeled energy consumption and greenhouse gas emissions. **d** Illustrative comparison of economic return, energy demand, greenhouse gas emissions and qualitative process descriptors

Defect-to-phase transduction is intended to preserve the functional value already embedded in S-LFP rather than recover Fe, P, and Li as separated chemical products. We therefore examined whether restoring cathode function provides a process-level advantage using the EverBatt 2023 model. Figure 5a compares the material and energy flows of pyrometallurgical recycling, hydrometallurgical recycling, and the proposed direct regeneration route. The pyrometallurgical route provides schematic process context [2], while the quantitative assessment is restricted to hydrometallurgy and direct regeneration for the modeled LFP feedstock.

The modeled economic results reflect both recovered-product revenue and processing costs. Direct regeneration is estimated to yield a net profit of US$2.28 kg^-1^ of spent LFP cell recycled, compared with US$0.22 kg^-1^ for hydrometallurgical recycling (Fig. 5b). The larger difference between the revenue bars than between the cost bars indicates that retaining LFP as a valuable cathode product is a major contribution to the profit advantage. The aggregated cost categories do not establish which individual chemical, wastewater or equipment cost contributes most to cost reduction. The energy and emission values are also expressed per kilogram of spent cell recycled. Direct regeneration requires 15.037 MJ kg^-1^ and produces 1.227 kg CO_2_ equivalent kg^-1^, whereas hydrometallurgy requires 18.362 MJ kg^-1^ and produces 1.536 kg CO_2_ equivalent kg^-1^ (Fig. 5c and Table S2). These values correspond to reductions of approximately 18% in energy demand and 20% in greenhouse gas emissions under the modeled conditions. Figure 5d provides an illustrative comparison, and Tables S3 and S4 document the material-price assumptions. These model-based estimates are conditional on the selected prices and process assumptions; they are not an industrial demonstration of the laboratory route. The improvements apply to total energy demand and greenhouse gas emissions, rather than to every environmental category in Table S2.

## Conclusions

This work examines defect-to-phase transduction together with carbon-interface reconstruction in the regeneration of spent LFP. Controlled oxidation converts the detectable olivine phase into Li_3_Fe_2_(PO_4_)_3_ and Fe_2_O_3_ phases while substantially attenuating the damaged carbon and binder signatures. Li compensation and reductive recombination then rebuild LFP from the identified intermediates. Along the complete regeneration route, the refined Fe-on-Li site occupancy decreases from 5.72% to 0.68%, showing substantial structural recovery in the guanidine-free material. The guanidine-assisted sample gives a lower estimated occupancy of 0.35% together with changes in carbon ordering and electrochemical response; these associations do not isolate a nitrogen-specific microscopic mechanism. Spatially resolved microscopy and spectroscopy show that the surface-to-bulk FP and Fe^3+^ gradients of S-LFP are replaced by a more coherent LFP lattice with a predominantly uniform Fe^2+^ state across the examined regions. The resulting cathode delivers 167 mAh g^-1^ at 0.1 C and 123 mAh g^-1^ at 5 C, while retaining 99.2% of its capacity after 500 cycles at 1 C. EverBatt analysis estimates a net profit of US$2.28 kg^-1^ of spent cell recycled, together with lower energy demand and greenhouse gas emissions than hydrometallurgical recycling. These results provide a comparative account of lattice recovery and guanidine-assisted interface reconstruction within oxidation-assisted regeneration, with mechanistic conclusions bounded by the available phase, occupancy and electrode-level measurements.

## Supplementary Information

Below is the link to the electronic supplementary material.

Supplementary file1 (DOCX 2365 kb)
