# Supplementary material for: Defect-to-Phase Transduction and Interface Reconstruction Enable Regeneration of Spent LiFePO4 Cathodes
Source: Nanomicro Lett. 2026 Nov 6;19:125. doi: 10.1007/s40820-026-02451-x (PMC13743707; doi:10.1007/s40820-026-02451-x)
Supplement: Supplementary file 1 [file 40820_2026_2451_MOESM1_ESM.docx]

Supporting Information for

**Defect-to-Phase Transduction and Interface Reconstruction Enable Regeneration of Spent LiFePO_4_ Cathodes**

Liwei Chen^1,2#^, Zhiheng Wu^1#^, Shouyu Qu^2^, Siyu Zhang^2^, Magdi E. A. Zaki^3^, Mohamed H. Helal^4^, Yangyang Liu^5^, Guanhua Jin^1^*, and Jiang Zhou^1,2^*

^1^ Xinjiang Key Laboratory of New Energy and Energy Storage Technology, Xinjiang Institute of Technology, Aksu, Xinjiang 843100, P. R. China

^2^ School of Materials Science and Engineering, Hunan Provincial Key Laboratory of Electronic Packaging and Advanced Functional Materials, Central South University, Changsha 410083, P. R. China

^3^ Department of Chemistry, Faculty of Science, Imam Mohammad Ibn Saud Islamic University (IMSIU), Riyadh 11623, Saudi Arabia

^4^ Center for Scientific Research and Entrepreneurship, Northern Border University, Arar 73213, Saudi Arabia

^5^ School of Instrument Science and Technology, Xi’an Jiaotong University, Xi’an 710049, P. R. China

#Liwei Chen and Zhiheng Wu contributed equally to this work.

*Corresponding authors. E-mail: [guanhuajin@xjit.edu.cn](mailto:guanhuajin@xjit.edu.cn) (Guanhua Jin); [zhou_jiang@csu.edu.cn](mailto:zhou_jiang@csu.edu.cn) (Jiang Zhou)

**S1 Supporting Figures**

**Fig. S1** **a)** XRD pattern of S-LFP; **b)** XRD pattern of S-LFP-Air.

**Fig. S2** High-resolution Fe 2p XPS spectra of **a)** S-LFP and **b)** S-LFP-Air.

**Fig. S3** Infrared spectroscopy analysis of S-LFP and S-LFP-Air.

**Fig. S4** TG-DTG curves of a) glucose and b) guanidine hydrochloride. The decomposition annotation is schematic rather than a stoichiometrically balanced reaction. TG alone does not identify the volatile decomposition products.

**Fig. S5** TG-DTG curves of R-LFP@CN precursor.

**Fig. S6** **a)** XPS spectra of S-LFP, R-LFP and R-LFP@CN; **b)** Fe 2p detailed XPS spectra of R-LFP and **c)** Fe 2p detailed XPS spectra of R-LFP@CN.

**Fig. S7** **a)** C 1s detailed XPS spectra of S-LFP, **b)** C 1s detailed XPS spectra of R-LFP and **c)** C 1s detailed XPS spectra of R-LFP@CN.

**Fig. S8** N 1s detailed XPS spectra of **a)** S-LFP and **b)** R-LFP.

Refinement interpretation. The Fe-on-Li values are model-derived occupancies in the refined olivine phase, rather than mass fractions of defects in the complete powder. GOF describes overall profile agreement and does not independently establish occupancy accuracy. The statistical significance of the difference between the refined Fe-on-Li occupancies of 0.68% and 0.35% cannot be established without occupancy uncertainties and an assessment of the refinement constraints.

**Fig. S9** **a)** XRD refinement diagram of S-LFP, **b)** XRD refinement diagram of R-LFP.

**Fig. S10** FT-IR spectra of S-LFP, R-LFP and R-LFP@CN.

**Fig. S11** Raman spectra of S-LFP, R-LFP and R-LFP@CN.

**Fig. S12** Electron microscopy images and corresponding EDS elemental maps of Fe, O and P for **a)** S-LFP and **b)** R-LFP@CN.

**Fig. S13** CV curves of R-LFP.

**Fig. S14** Contour plot of cyclic voltammograms at different scan rates of R-LFP.

**Fig. S15** In-situ EIS spectra of S-LFP, R-LFP and R-LFP@CN.

**Fig. S16** In situ DRT profiles of R-LFP.

**Fig. S17** Charge–discharge profiles of R-LFP at 1 C during the 1st, 300th and 500th cycles.

**Table S1** Bulk molar ratios of Li, Fe and P in S-LFP and R-LFP@CN.

| Molar ratios | Li | Fe | P |
| --- | --- | --- | --- |
| S-LFP | 81.2 | 100.0 | 96.1 |
| R-LFP@CN | 104.0 | 100.0 | 96.6 |

Note: Ratios are normalized to Fe = 100. The measured bulk Li/Fe and P/Fe ratios are not an exact 1:1:1 composition. Li replenishment is evident, but these results and the identified crystalline phases do not establish a complete intermediate-phase or elemental mass balance. XRD phase purity should not be equated with exact bulk stoichiometry.

**Table S2** Life-cycle environmental impacts of different recycling methods.

|  | Hydro | Direct |
| --- | --- | --- |
| **Total energy use in MJ per kg cell recycled** |  |  |
| Total energy | 18.362 | 15.037 |
| Fossil-fuels | 17.190 | 12.893 |
| Coal | 5.364 | 10.373 |
| Natural gas | 5.270 | 1.597 |
| Water use in gallon | 6.556 | 0.922 |
| **Total Emission in g per kg cell recycled** |  |  |
| VOC | 0.418 | 0.131 |
| CO | 1.746 | 0.549 |
| NO_x_ | 4.438 | 1.119 |
| PM10 | 0.283 | 0.209 |
| PM2.5 | 0.228 | 0.116 |
| SO_x_ | 0.876 | 1.186 |
| BC | 0.127 | 0.008 |
| OC | 0.046 | 0.016 |
| CH_4_ | 2.329 | 1.919 |
| N_2_O | 0.032 | 0.023 |
| CO_2_ | 1,454 | 1,162 |
| CO_2_ (w/C in VOC & CO) | 1,458 | 1,164 |
| GHGs | 1,536 | 1,227 |

Note: Energy use and emission values are model-based estimates from EverBatt 2023, expressed per kilogram of spent cell recycled. They depend on the selected model assumptions and do not represent measurements from an industrial implementation of the laboratory process.

**Table S3** The price of input materials.

| The price of materials | | | | |
| --- | --- | --- | --- | --- |
| No | item | Market price | unit | Data source |
| 1 | Spent LFP batteries | 1 | $ kg^-1^ | https://hq.smm.cn(SMM) |
| 2 | H_2_SO_4_ | 0.24 | $ kg^-1^ | https://www.100ppi.com/ppi(100PPI) |
| 3 | H_2_O_2_ | 0.11 | $ kg^-1^ | 100PPI |
| 4 | Na_2_CO_3_ | 0.17 | $ kg-^1^ | 100PPI |
| 5 | Li_2_CO_3_ | 19.03 | $ kg^-1^ | 100PPI |
| 6 | Glucose | 0.42 | $ kg^-1^ | 100PPI |
| 7 | CH_6_ClN_3_ | 1.46 | $ kg^-1^ | https://china.guidechem.com |
| 8 | Water | 0.0019 | $ gallon^-1^ | https://www.h2o-china.com |
| 9 | Electricity | 0.07 | $ kWh^-1^ | https://m.bendibao.com |
| Update time: 2026/8/11 | | | | |

**Table S4** The price of recycled materials.

| The price of recycled materials | | | | |
| --- | --- | --- | --- | --- |
| No | item | Market price | unit | Data source |
| 1 | LiFePO_4_ | 7.93 | $ kg^-1^ | SMM |
| 2 | Graphite | 4.10 | $ kg^-1^ | SMM |
| 3 | Al foil | 3.33 | $ kg^-1^ | SMM |
| 4 | Cu foil | 15.01 | $ kg^-1^ | SMM |
| 5 | Lithium carbonate | 12.75 | $ kg^-1^ | SMM |
| 6 | Steel | 0.42 | $ kg^-1^ | SMM |
| 7 | Plastics | 0.66 | $ kg^-1^ | SMM |
| Update time: 2026/8/11 | | | | |
